# The Historical Evolution of Microlearning in Health Professions Education: Bibliometric Analysis

**DOI:** 10.2196/91616

**Published:** 2026-06-01

**Authors:** Jennie C De Gagne, Paige S Randall, Elizabeth R Blackwood, Katherine A Carlson, Hee Sun Kang

**Affiliations:** 1 School of Nursing, Duke University, Durham, NC, United States; 2 School of Nursing, University of North Carolina, Chapel Hill, NC, United States; 3 Medical Center Library & Archives, Duke University, Durham, NC, United States; 4 Red Cross College of Nursing, Chung-Ang University, 84, Heukseok-Ro, Dongjak-Gu, Seoul, 06974, Republic of Korea, 82 2-820 ext 5699

**Keywords:** artificial intelligence, AI, bibliometrics, health professions education, microlearning, mobile learning

## Abstract

**Background:**

Microlearning has emerged as a flexible, technology-enabled instructional approach in health professions education, yet its longitudinal evolution, collaboration patterns, and thematic development remain underexplored.

**Objective:**

This study aimed to map publication trends, research impact, collaboration networks, and thematic evolution of microlearning research within health professions education.

**Methods:**

A bibliometric analysis of the Web of Science Core Collection (1977‐2025) was conducted. Records were retrieved, screened, cleaned, and analyzed using established bibliometric techniques to examine publication trends, coauthorship networks, and keyword co-occurrence patterns.

**Results:**

A total of 560 publications were included in the analysis. Publications demonstrated substantial growth beginning in 2015, with the greatest expansion after 2020. Research output was concentrated in English-speaking regions, particularly the United States. Coauthorship networks showed moderate collaboration within several established academic hubs. Keyword analyses revealed 5 thematic domains reflecting the field’s progression from instructional efficiency toward mobile, social, simulation-enhanced, and crisis-responsive microlearning. These themes suggest increasing attention to experiential, accessible, and adaptive learning approaches within health professions education.

**Conclusions:**

Microlearning has evolved into a multidimensional scholarly area shaped by digital transformation, shifting pedagogical expectations, and increased demand for flexible learning formats. Ongoing development should prioritize global representation, theoretical grounding, and rigorous evaluation of emerging technologies. The findings may inform educators, curriculum designers, and institutional leaders seeking to implement evidence-based, digitally enabled microlearning strategies that support learners across diverse educational and clinical environments.

## Introduction

The integration of technology into health professions education (HPE) has fundamentally reshaped how knowledge is delivered and acquired, aligning with broader transitions toward flexible, learner-centered educational paradigms [1,2]. Within this digital transformation, microlearning—characterized by concise, focused, technology-mediated content—has emerged as an effective strategy to enrich HPE [3,4]. Defined as “bite-sized,” “just-in-time,” or “on-demand” learning, microlearning leverages digital platforms to provide efficient, self-directed training for students, educators, and practitioners, meeting the field’s growing demand for rapid knowledge updates and sustained clinical competency [3]. Artificial intelligence (AI) further enhances the feasibility of microlearning by streamlining design, development, and implementation processes [5], positioning microlearning as a dynamic and scalable component of online learning to foster positive cognitive, behavioral, and affective outcomes [6].

Despite growing interest, the scope, evolution, and scholarly influence of microlearning in HPE remain underexamined. A foundational scoping review by De Gagne et al [4] demonstrated microlearning’s potential across multiple health professions. That review highlighted microlearning’s role in improving learner engagement and retention through succinct, interactive formats, particularly when integrated with digital or adaptive technologies. Cronin and Durham [3] further conceptualized microlearning as a “self-directed, on-demand teaching strategy” characterized by focus, brevity, and multimodal delivery, although they emphasized the absence of definitional consistency across studies.

Bibliometric investigations in HPE have increasingly mapped research on technology-enhanced education, including online learning [7-9], mobile learning [10], and simulation [11-14]. Simulation-based learning has received significant scholarly attention [15], and social media–enabled learning is gaining traction as an engagement-driven modality [16]. Within this expanding digital landscape, microlearning appears frequently as an instructional strategy that delivers short, targeted content across formats such as videos, quizzes, podcasts, and social media clips. However, existing reviews are largely narrative or scoping in nature and often limited in focus. For example, one systematic review evaluated simulation-based microlearning for health care professionals [17], another focused on mobile microlearning in nursing professional development [18], and a literature review examined microlearning in combination with social media across select disciplines [19]. These studies demonstrate microlearning’s versatility and growing relevance, yet they do not provide a comprehensive, quantitative overview of its development, adoption patterns, and intellectual structure within HPE.

Given the rapid expansion of microlearning-related methods, there is a need to systematically map its scholarly trajectory. Bibliometric analysis offers a quantitative approach for examining research productivity, citation impact, collaborative structures, and thematic evolution over time [20]. Accordingly, this study conducted a bibliometric analysis of microlearning in HPE to (1) analyze publication trends and citation impact (eg, annual output, journal distribution, geographic output, and influential references); (2) examine collaboration networks at the author, institutional, and national level; and (3) explore keyword co-occurrences to identify prevailing themes and emerging areas of focus.

## Methods

### Overview

This study used a longitudinal bibliometric analysis to examine the evolution and characteristics of microlearning within HPE. Bibliometric analysis enables quantitative evaluation of scholarly activity by generating publication, citation, and collaboration metrics through specialized software [21]. The study protocol was registered on the Open Science Framework to promote transparency and reproducibility [22].

### Ethical Considerations

Ethics approval was not required as only publicly available bibliometric data were analyzed.

### Data Collection and Cleaning

Data were retrieved from the Web of Science (WoS) Core Collection due to its comprehensive coverage of high-impact scholarly publications. A search strategy developed by professional medical librarians in collaboration with the author team was executed on June 11, 2025. Search filters were applied in the WoS user interface to limit results to the document types “article” and “review article,” yielding 9552 records. The search incorporated terms related to HPE, specific health professions, and microlearning. To ensure comprehensive retrieval, we operationalized microlearning and microlearning-adjacent terms (eg, “microteaching,” “nano-learning,” and “just-in-time learning”) as instructional approaches involving brief, focused learning units often delivered through digital or modular formats. These terms were treated as conceptually adjacent or antecedent instructional approaches rather than direct equivalents of contemporary microlearning. Studies using any of these terms were included only when the described intervention aligned with our operational definition of microlearning and met all other eligibility criteria. The complete search strategy is available in Table S1 in Multimedia Appendix 1.

Records were imported into Covidence (Veritas Health Innovation) [23] for deduplication and title and abstract screening. Screening was conducted independently by 2 reviewers using predefined eligibility criteria (Table 1), with discrepancies resolved by a third reviewer. Microlearning alignment was determined during title and abstract screening based on evidence that the instructional approach was brief, focused, and delivered in modular or segmented formats consistent with microlearning principles. Because this study was designed as a bibliometric analysis, screening focused on relevance and eligibility based on bibliographic metadata, titles, abstracts, and indexed record information instead of the conventional full-text appraisal used in evidence synthesis reviews. To restore any bibliometric metadata not retained during Covidence import, full citation details were re-extracted from WoS using accession numbers. Data were then imported into OpenRefine [24] for metadata standardization. Author names, institutional affiliations, and keywords were cleaned and consolidated to prevent duplication and ensure analytical consistency. No additional records were excluded during this phase; however, document types were verified and corrected as necessary. The final dataset comprised 560 studies for analysis. A flowchart based on the PRISMA (Preferred Reporting Items for Systematic Reviews and Meta-Analyses) guidelines [25] is provided in Figure S1 in Multimedia Appendix 1. The eligibility criteria table presents the full set of prespecified inclusion and exclusion criteria, whereas the flow diagram summarizes the major exclusion categories applied during title and abstract screening. Individual exclusion reasons were not itemized for each excluded record.

**Table 1.** Eligibility criteria.

Domain	Inclusion criteria	Exclusion criteria
Population	Students, trainees, or licensed professionals engaged in HPE^a^ (eg, nursing, medicine, pharmacy, public health, allied health, dentistry, physical therapy, and occupational therapy)	Populations not relevant to microlearning in HPE: studies focused on patient or caregiver education or other nonlearner populations, health-related training outside formal educational or professional development settings (eg, community outreach or corporate wellness), and veterinary education
Intervention or exposure	Studies focused on microlearning or microlearning-adjacent instructional strategies (eg, bite-sized learning, just-in-time learning, mobile microcontent, or nano-learning)	Studies lacking a clear microlearning or microlearning-adjacent instructional focus
Context	HPE settings, including academic, clinical, and continuing professional development environments	—^b^
Study characteristics	Scholarly publications retrieved from WoS^c^ and initially filtered as “article” or “review article” published in any language and year	Non–peer-reviewed publication types (eg, editorials, letters, or commentaries), study protocols, or preregistrations

a HPE: health professions education.

b Not applicable.

c WoS: Web of Science.

### Data Analysis

Bibliometric analyses were conducted using Biblioshiny (K-Synth Srl) [26] and VOSviewer (Centre for Science and Technology Studies, Leiden University) [27] to evaluate publication trends, citation impact, geographic output, collaboration networks, and thematic structures. Descriptive statistics, including annual publication counts and citation summaries, were compiled in Microsoft Excel for supplementary reporting. Biblioshiny, a web-based interface of the *bibliometrix* R package [26], facilitated descriptive analyses of publication trajectories, citation metrics, global contributions, influential authors and institutions, and cocitation networks to reveal the intellectual structure of microlearning research in HPE. In VOSviewer, collaboration networks were visualized at the author, institutional, and national levels, and keyword co-occurrence analysis using author-assigned keywords was conducted to identify thematic clusters and emerging areas of focus [28,29]. Full counting and association strength normalization were used to construct the maps. Thresholds were applied to reduce network complexity and improve interpretability, with minimum occurrence and document thresholds set according to the unit of analysis, and only connected items were retained in the final visualizations. The resulting maps displayed cluster membership, number of links, and total link strength (TLS) for included items. Entity labels in VOSviewer figures reflect the indexed source metadata and software parsing conventions used at the time of analysis. Additional analytical details are provided in Table S2 in Multimedia Appendix 1.

## Results

### Publication Trends and Citation Impact

The final dataset comprised 560 publications indexed between 1977 and 2025 authored by 2604 unique contributors and citing 13,229 references, with an average of 4.86 (SD 2.80) coauthors per publication. Nearly all publications (n=549, >98%) were in English, with only a small number in other languages. Most documents (n=514, 91.8%) were full research articles, followed by review articles (n=41, 7.3%), proceedings papers (n=4, 0.7%), and book chapters (n=1, 0.2%). Although the WoS filter was limited to “article” and “review article,” document type metadata in the final dataset included a small number of records labeled as proceedings papers or a book chapter following export and metadata verification. Metadata completeness was high, although inconsistencies in author keywords (n=115, 20.5%) and KeyWords Plus (n=136, 24.3%) warrant caution when interpreting keyword-based trends (Table 2).

Publication activity was minimal prior to 2005, after which output increased gradually until 2014 (1‐11 publications per year). A stronger upward trajectory was observed between 2015 and 2019 (16‐29 publications annually), followed by a marked surge from 2020 onward, peaking at 93 publications in 2024. As of the search date, 7.3% (41/560) of the publications had already been indexed in 2025, indicating sustained momentum. The calculated annual growth rate was 8.04%. Citation concentrations appeared to cluster around publications from years with higher output, although these patterns should be interpreted cautiously because citation counts are cumulative and time dependent.

**Table 2.** Summary of dataset characteristics from Biblioshiny.

Category	Values
Total documents, n	560
Time span	1977-2025
Sources (eg, journals), n	266
Annual growth rate (%)	8.04
Document age (y), mean (SD)	4.81 (4.69)
References cited, n	13,229
Authors, n	2604
Single-authored documents, n	31
Coauthors per document, mean (SD)	4.86
Keywords (DE^a^)^b^, n	1312
KeyWords Plus (ID)^c^, n	638

a DE: descriptors (author keywords).

b Provided by study authors.

c Generated/indexed keywords from databases.

### Journal and Source Distribution

Among the 560 publications, 514 (91.8%) research articles were distributed across 266 journals, indicating broad and growing interest in microlearning across diverse HPE contexts. The most productive journal was *BMC Medical Education* (43/514, 8.4%), followed by *Anatomical Sciences Education* (17/514, 3.3%), *Nurse Education Today* (15/514, 2.9%), *Medical Science Educator* (13/514, 2.5%), and *Nurse Education in Practice* (11/514, 2.1%). Three journals—*Journal of Dental Education*, *Journal of Nursing Education*, and *Journal of Surgical Education*—contributed 1.9% (10/514) of the publications each. Additional frequently publishing journals included *Currents in Pharmacy Teaching and Learning* (9/514, 1.8%), *Medical Teacher* (9/514, 1.8%), *Nurse Educator* (9/514, 1.8%), and *Academic Medicine* (8/514, 1.6%). Citation trends mirrored these publication patterns, with the most frequently cited articles often appearing in the journals and years with peak research activity.

### Geographic and Institutional Productivity

Microlearning research demonstrated broad international representation, although output was concentrated in high-resource regions. The United States was the leading contributor (219/560, 39.1%), followed by the United Kingdom (46/560, 8.2%), China (38/560, 6.8%), Canada (32/560, 5.7%), and India (28/560, 5%). Additional contributions from multiple regions suggest expanding global engagement with microlearning in digital and flexible health professions training environments. Institutional productivity was similarly concentrated among leading academic centers. Harvard University (43/560, 7.7%), the University of Toronto (40/560, 7.1%), and the University System of Ohio (35/560, 6.3%) were the most prolific, followed by the University of California system (22/560, 3.9%) and Johns Hopkins University (21/560, 3.8%). This pattern indicates that established academic hubs have driven early scholarship, with increasing participation emerging worldwide across additional institutions.

### Intellectual Influence: Most Cited References

Citation impact analysis identified seminal works that shaped early discourse on microlearning and digitally mediated learning. The most frequently cited reference was the work by De Gagne et al [4] (n=36 citations), a scoping review synthesizing microlearning modalities and outcomes. Cho et al [30] (n=29 citations) examined the educational value of podcasts, whereas Mallin et al [31] (n=23 citations) documented the adoption of asynchronous digital learning resources in medical residency education. Additional influential publications included those by Cheston et al [32] and Riddell et al [33] (n=22 citations each), which reinforced the role of mobile, social, and podcast-based learning. Collectively, these works demonstrated that early scholarship positioned microlearning within broader digital, mobile, and networked learning ecosystems.

### Coauthorship Networks: Research Collaboration Networks

#### Overview

Coauthorship networks were mapped using VOSviewer with full counting and association strength normalization. All networks were generated directly from the study dataset and reflect bibliometric relationships within the included publications. Thresholds were applied, and only connected items were retained in the final visualizations.

#### Author-Level Collaboration

Authors with at least one document were eligible for inclusion in the coauthorship analysis. Thresholds were applied, and only connected items were retained in the final visualization, resulting in 32 authors. Three distinct coauthorship clusters emerged, corresponding to major collaborative teams within the field (cluster 1=red; cluster 2=green; cluster 3=blue; Figure S2 in Multimedia Appendix 1). TLS quantified the intensity of coauthorship connections among authors. Nematollahi exhibited the highest TLS [21], followed by Walker and Nolan (TLS=13 each), serving as key connectors within and between their respective clusters. Overall, collaboration intensity was moderate, with most publications produced by small- to mid-sized author groups rather than large-scale, multi-institutional teams (Figure S2 in Multimedia Appendix 1).

#### Institutional and National Collaboration

The final institutional coauthorship visualization displayed 39 institutions. Six distributed collaboration clusters emerged, indicating several institution-level partnership groupings within the field. The University of Michigan showed the strongest institutional linkage (TLS=14), followed by Johns Hopkins University; the University of California, San Francisco; and the University of Alabama at Birmingham (TLS=13 each). Institutions such as Emory University (TLS=12) and the University of Maryland (TLS=10) contributed to cross-cluster linkages. The network structure reflects multiple research hubs rather than single-institution dominance (Figure S3 in Multimedia Appendix 1).

**Figure 1.**
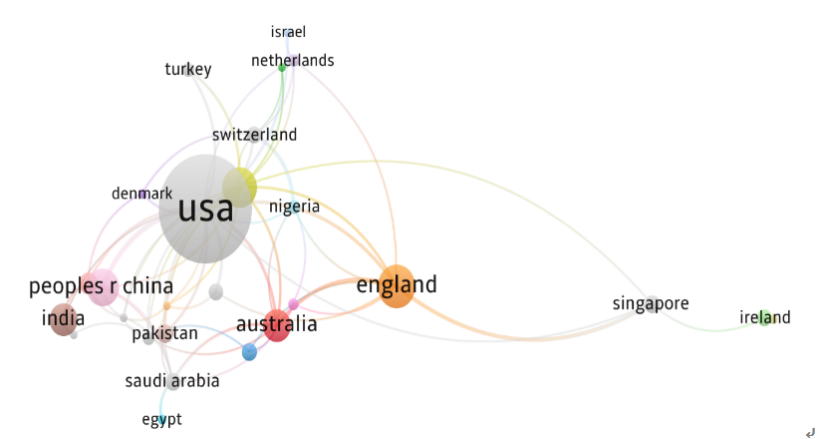
National coauthorship network in microlearning research within health professions education. Node size reflects total publications, and line thickness indicates collaboration intensity (total link strength). Proximity between nodes represents coauthorship frequency, and color clusters denote distinct regional or collaborative groupings. Thresholds were applied to reduce network complexity, and only connected items were retained in the visualization. Country labels in the VOSviewer map reflect source metadata and software-based parsing conventions and, therefore, may differ slightly from the standardized geopolitical naming used in the manuscript text.

At the national level, the final coauthorship visualization displayed 27 countries. The country network showed several interconnected collaboration groupings, with a small number of countries serving as major hubs. The United States served as the central hub (46 links; TLS=239), with strong collaborative ties to Canada, the United Kingdom, Australia, and China. Canada (TLS=28) and the United Kingdom (TLS=18) functioned as secondary anchors bridging networks across North America, Europe, and Asia-Pacific regions. Other countries contributed with lower TLS values (≤3), indicating emerging rather than sustained collaboration. Overall, microlearning research demonstrated a predominantly US-centered network with emerging global participation (Figure 1).

### Keyword Co-Occurrence and Thematic Trends

**Figure 2.**
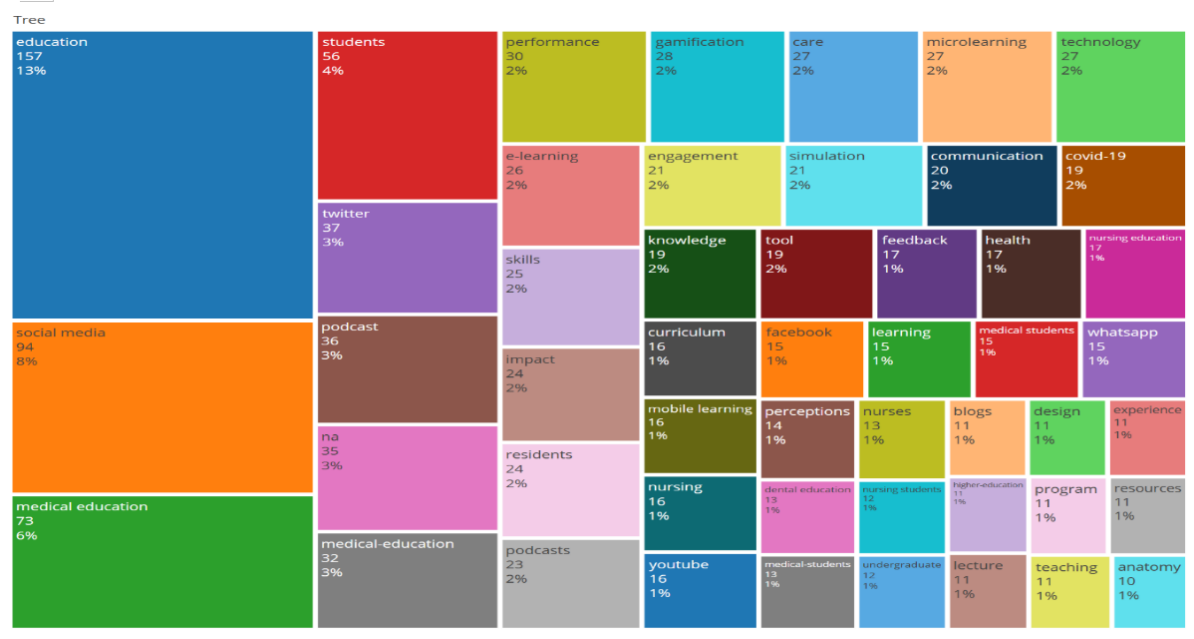
Tree map of the most frequent keywords in the microlearning literature within health professions education. Rectangle size is proportional to keyword frequency, with larger blocks indicating more frequently occurring terms in the dataset.

**Figure 3.**
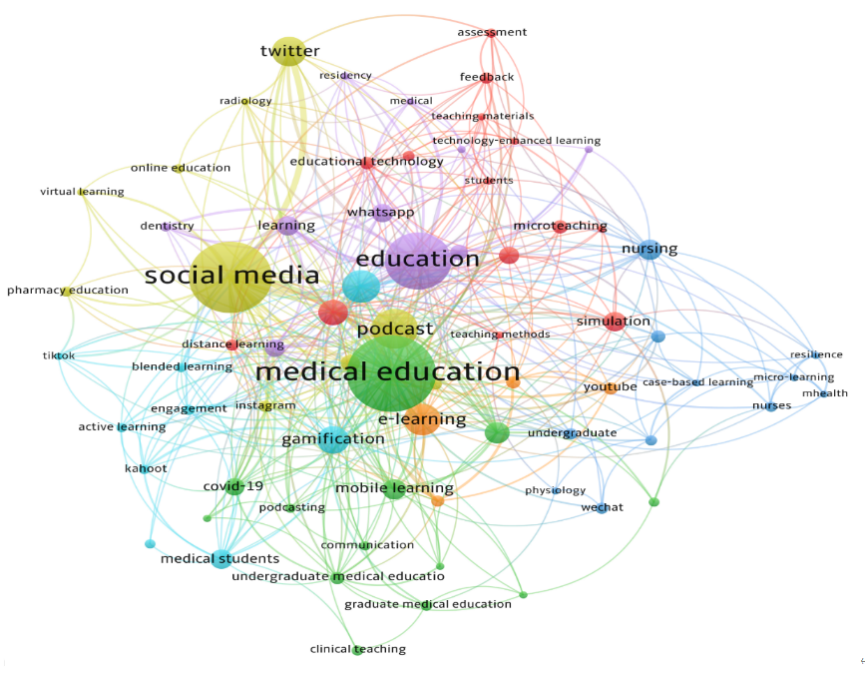
Keyword co-occurrence network map of microlearning research in health professions education. Nodes represent keywords, with larger nodes indicating higher frequency of occurrence. Lines represent co-occurrence relationships, with thicker lines indicating stronger connections. Colors denote the 7 distinct keyword clusters identified by VOSviewer. Thresholds were applied to reduce network complexity, and the final visualization displays 67 connected author keywords, which were subsequently synthesized into 5 higher-order thematic domains for interpretation in the text.

**Figure 4.**
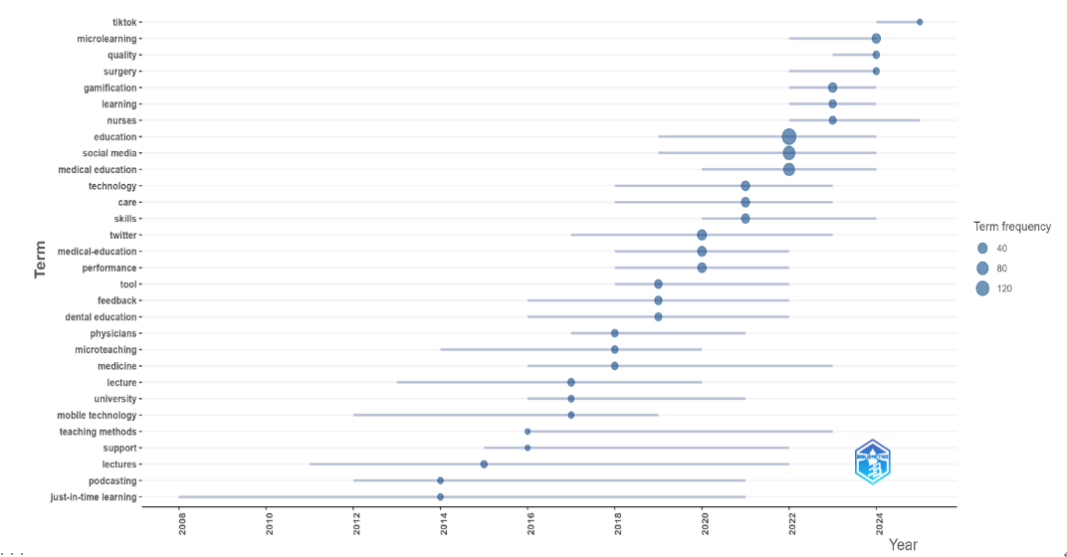
Temporal distribution of trending keywords in microlearning research within health professions education. Each bubble represents a keyword, positioned according to its average year of occurrence in the dataset. Bubble size reflects term frequency, and horizontal lines indicate the span of years during which each term appeared, highlighting the shift from foundational instructional concepts to more digitally integrated learning modalities over time.

Keyword frequency analysis identified recurrent concepts, with “education” (n=157), “social media” (n=94), “medical education” (n=73), “students” (n=56), and “podcast” (n=36) among the most common (Figure 2). The VOSviewer co-occurrence network revealed 7 keyword clusters, which were synthesized into five higher-order thematic domains to enhance interpretability: (1) digital and mobile microlearning in medical education (clusters 1 and 7), (2) social media–driven learning and engagement (cluster 2), (3) pedagogical strategies and assessment (cluster 3), (4) simulation-based skill development in nursing and allied health (cluster 4), and (5) crisis-driven learning adaptation and performance optimization (clusters 5 and 6; Figure 3). Temporal mapping demonstrated an evolution from foundational instructional concepts (eg, lecture, microteaching, and just-in-time learning) toward more digitally integrated modalities (eg, podcasts, simulation, mobile learning, gamification, and TikTok; Figure 4).

## Discussion

### Growth and Maturation of Microlearning

Microlearning in HPE has evolved significantly over nearly 5 decades, progressing from sporadic experimentation to a widely recognized pedagogical approach. The earliest publications identified in this dataset appeared in 1977 (Crosby [34]) and 1989 (Hargie and Morrow [35]), reflecting early instructional approaches such as microteaching and microtraining that share features with contemporary microlearning. These studies can be interpreted as antecedent or conceptually related forms rather than direct representations of modern microlearning as currently defined in digitally mediated educational contexts. Growth accelerated after 2015 and surged following 2020, reflecting broader shifts toward flexible, competency-based digital learning and the need for rapid, time-efficient knowledge acquisition during pandemic-driven transitions. These patterns suggest that microlearning has become increasingly embedded within contemporary expectations for modular, accessible, and self-directed learning. Because citation counts accumulate over time, comparisons across publication years should be interpreted cautiously and understood as descriptive indicators rather than direct measures of relative scholarly influence.

The dominance of English-language publications and concentration of research in high-resource regions indicate that knowledge production remains unevenly distributed. While this pattern likely reflects existing academic infrastructures for digital pedagogy, it also points to inequities in representation and visibility for low- and middle-income contexts, where innovation may be occurring but less frequently indexed. Increasing citation activity during peak publication years may suggest growing scholarly attention and thematic convergence within the field, although bibliometric indicators alone cannot confirm scholarly consolidation. Foundational work on digitally mediated learning appears to have supported subsequent research on the design, implementation, and evaluation of microlearning across diverse HPE settings. Collectively, these patterns suggest that microlearning is becoming an increasingly established area of inquiry shaped by evolving technological ecosystems and the demand for scalable, adaptive instructional strategies.

### Collaboration Patterns and Knowledge Flow

Collaboration patterns provide insights into how microlearning knowledge is generated and disseminated worldwide. The author-level network demonstrated moderate collaboration intensity, indicating that research is largely conducted within small, localized teams rather than large, internationally integrated consortia, similar to recent literature [7,36]. A limited number of recurring authors served as central connectors, shaping conceptual trajectories, supporting methodological consistency, and facilitating knowledge diffusion across groups.

Institutional collaboration patterns reflect a distributed, multi-hub research structure rather than dominance by a single academic center. Several well-established North American institutions functioned as collaborative anchors, with additional but more modest engagement from European universities. This distribution suggests both a regional concentration of leadership and the gradual development of research capacity within secondary academic settings.

At the national level, the United States emerged as the central hub of global collaboration, with the United Kingdom, Canada, Australia, and China functioning as secondary contributors. Emerging participation from regions such as the Middle East, South Asia, and parts of Europe suggests increasing geographic diversification. As the field continues to mature, broader inclusion of diverse institutional and cultural perspectives may enhance contextual relevance and expand the adaptability of microlearning models across varied educational environments.

### Thematic Evolution and Pedagogical Directions

The thematic progression of microlearning research indicates a clear pedagogical evolution within HPE. Early clusters associated with instructional efficiency suggest that microlearning initially emerged as a strategy to fragment content delivery for rapid recall and time-constrained knowledge acquisition. As mobile and digital platforms gained prominence, the focus shifted toward learner autonomy, self-regulation, and continuous access, reflecting broader trends in competency-based and personalized education [37].

A subsequent phase emphasized social media–driven microlearning, in which learning appeared increasingly peer mediated, networked, and embedded in informal digital ecosystems. This pattern may reflect growing attention to learner engagement, identity formation, and community participation as potentially important dimensions of microlearning. More recently, crisis-responsive and resilience-oriented themes appear more frequently in the literature, particularly during the COVID-19 pandemic [38,39]. Recent keyword patterns also indicate growing attention to themes such as resilience and emotional regulation, although these observations should be interpreted as descriptive patterns in term use rather than definitive evidence of thematic shift [4,40]. Collectively, these patterns suggest a broadening of microlearning scholarship beyond content reduction toward a more multidimensional pedagogical framing that includes accessibility, engagement, adaptability, and affective support.

The thematic domains identified in this study relate to established learning theories or frameworks. For example, digital and mobile microlearning delivers short, focused information segments that may minimize cognitive load, aligning with cognitive load theory. Social media–driven learning may enhance autonomy, allowing learners to engage in and regulate their learning while interacting with peers, consistent with self-regulated learning principles. In addition, simulation-based microlearning may support confidence development by enabling learners to practice tasks in simulated scenarios. Crisis-driven learning may reflect principles of situated learning by supporting knowledge development through contextually grounded participation in authentic or practice-relevant environments. These approaches may also provide contextualized practice opportunities for skill development aligned with competency-based education frameworks [6,41-44]. These connections suggest that microlearning functions not only as a technological format but also as a pedagogically grounded learning approach.

### Implications and Future Directions

The maturation of microlearning in HPE presents key implications for curriculum design, faculty development, and learner engagement. As competency-based and outcome-driven models increasingly prioritize rapid knowledge acquisition and performance reinforcement, microlearning offers modular, time-efficient formats that can be integrated across didactic, simulation-based, and workplace learning [45]. In competency-based pathways, microlearning may support adaptive progression by targeting individual performance gaps and sequencing content responsively.

The rise of mobile, social, and simulation-enhanced microlearning underscores its utility for experiential, just-in-time learning beyond classroom settings. Scenario-based formats can reinforce clinical reasoning and decision-making while supporting continuing professional development and rapid dissemination of practice updates [17]. However, meaningful integration requires pedagogical alignment; poorly structured microcontent risks fragmented knowledge acquisition rather than deep learning. Therefore, faculty development must extend beyond content creation to include sequencing, scaffolding, reflection, and alignment with assessment and competency frameworks. Moreover, as socially driven and resilience-oriented microlearning increases, concerns emerge regarding digital credibility, professionalism, cognitive overload, and learner well-being [40]. Thus, microlearning should be viewed not only as an instructional method but also as part of broader discussions on digital citizenship and how learners construct professional identity within networked environments.

Research on AI-driven adaptive microlearning represents a key frontier. Generative AI may enable dynamic scenario generation, real-time feedback, and performance-based content sequencing. Investigating its effectiveness, cognitive implications, and ethical risks will be crucial as adoption increases [46]. Similarly, immersive microlearning through virtual or augmented reality warrants exploration, particularly for supporting emotional regulation, identity formation, and resilience in demanding clinical environments. Finally, studies examining faculty readiness, institutional integration, and sustainability will support scalable adoption. Cross-disciplinary collaboration among educators, technologists, cognitive scientists, and AI experts will be essential to fully realize microlearning’s potential as a transformative and equitable educational modality in HPE.

These findings have implications across multiple levels of HPE. At the curricular level, microlearning can be incorporated into existing courses through short, focused learning modules (eg, brief videos, quizzes, or case-based microcontent) to support learner engagement and knowledge retention [4,47]. In faculty development, educators may benefit from training in designing and implementing microlearning activities and in using digital technologies effectively [48]. At the institutional level, leaders can support implementation by providing microlearning platforms or integrating them into existing learning management systems while also evaluating learning outcomes and supporting continuous improvement based on feedback.

### Strengths and Limitations

This study’s major strength lies in its comprehensive and systematically executed search strategy, which initially identified nearly 10,000 records through the WoS Core Collection. Collaboration with professional medical librarians ensured methodological rigor, transparent inclusion criteria, and reproducibility throughout the review process.

Several limitations should be acknowledged. Reliance on a single database may have restricted the retrieval of relevant studies indexed elsewhere, particularly those outside English-language or biomedical education outlets. Although the inclusion criteria did not impose language restrictions, the final dataset was overwhelmingly in English, reflecting both indexing practices and broader publication inequities. Other major databases offer complementary coverage. For instance, Scopus provides broader international journal representation, PubMed extensively indexes biomedical literature, and ERIC focuses on education-related publications. The exclusion of these databases may have influenced geographic representation, disciplinary breadth, and the inclusion of education-focused journals not indexed in WoS.

Additionally, metadata inconsistencies in author keywords and KeyWords Plus fields, which are common in bibliometric datasets, may limit the interpretive depth of keyword co-occurrence analyses. Potential inconsistencies between author keywords and KeyWords Plus may have influenced theme emergence, clustering stability, and the interpretation of temporal trends. For example, evolving topics may have been labeled differently over time, which could affect how related studies were grouped and how thematic shifts were interpreted. The inclusion of microlearning-adjacent or antecedent terms (eg, “microteaching” and “microtraining”) may also have influenced the historical framing of the field by capturing earlier instructional approaches that share structural similarities with contemporary microlearning but differ in technological context and pedagogical framing. A sensitivity check excluding the 2 antecedent records from 1977 and 1989 showed no meaningful changes in keyword co-occurrence patterns or thematic interpretation, supporting the robustness of the findings. The search strategy also included a broad set of mobile- and social media–related instructional terms to ensure comprehensive retrieval. Although screening retained only studies aligned with the operational definition of microlearning, this approach may have broadened the thematic scope of the dataset, contributing to the prominence of social media–related keywords in the results. These factors should be considered when interpreting the findings. In addition, the bibliometric approach captures quantitative trends rather than the pedagogical quality or contextual nuance of the included studies, underscoring the need for complementary qualitative and theoretical reviews.

### Conclusions

Microlearning in HPE has evolved from a fragmented instructional strategy into a multidimensional and increasingly globalized area of inquiry. This bibliometric analysis demonstrates a rapidly expanding field shaped by digital transformation; collaborative networks; and shifting pedagogical priorities aligned with mobility, social engagement, emotional resilience, and adaptive learning. As microlearning matures, future research should prioritize theoretical integration, global inclusivity, faculty readiness, and the pedagogical potential of AI-driven adaptive technologies. These directions will be essential for sustaining microlearning’s impact in preparing current and future health professionals for dynamic, digitally mediated clinical environments.

## Supplementary material

10.2196/91616 Multimedia Appendix 1 Search strategy, bibliometric procedures, and coauthorship networks.

## Abbreviations

AI: artificial intelligence
HPE: health professions education
PRISMA: Preferred Reporting Items for Systematic Reviews and Meta-Analyses
TLS: total link strength
WoS: Web of Science
