# Supplementary material for: The Historical Evolution of Microlearning in Health Professions Education: Bibliometric Analysis
Source: JMIR Med Educ. 2026 Jun 1;12:e91616. doi: 10.2196/91616 (PMC13225502; doi:10.2196/91616)
Supplement: Multimedia Appendix 1 [file mededu-v12-e91616-s001.docx]

**Multimedia Appendix 1**

**Table S1.** Search strategy

**Librarian Searcher**: Katherine Carlson, MLIS; Duke University Medical Center Library & Archives, Duke University School of Medicine

**Peer Review of Search**: Elizabeth Blackwood, MSLS; Duke University Medical Center Library & Archives, Duke University School of Medicine

**Date of original search**: 6/11/2025

**Database: Web of Science Core Collection (via Clarivate)**

| **Search** | **Strategy** | **Results** |
| --- | --- | --- |
| #1 Health Professions Education | TS=("medical student*" OR "nursing student*" OR (("medical faculty" OR "allied health" OR chiropractic OR dental OR midwifery OR medical OR Nursing OR pharmacy OR "public health" OR podiatry OR “occupational therapy” OR “occupational therapist” OR “occupational therapists” OR “occupational sciences” OR “physical therapy" OR "physical therapist" OR "physical therapists" OR physiotherapy OR physiotherapies OR physiotherapist OR physiotherapists) AND (education OR faculty OR school OR schools OR student OR students OR trainee OR trainees OR fellow OR fellows OR resident OR residents OR university OR universities))) | 614,810 |
| #2 Health Professionals | TS=(“health care professional” OR "health care professionals” OR “medical faculty" OR “dental faculty” OR “nursing faculty” OR "allied health" OR “allied health personnel” OR “healthcare practitioner” OR nurses OR nurse OR nursing OR “nurse practitioner” OR “nurse practitioners” OR “practice nurse” OR “practice nurses” OR chiropractor OR chiropractors OR dentist OR dentists OR optometrist OR optometrists OR osteopath OR osteopaths OR midwife OR midwives OR pharmacist OR pharmacists OR psychologist OR psychologists OR "public health" OR podiatrist OR podiatrists OR “occupational therapist” OR “occupational therapists” OR "physical therapist" OR "physical therapists" OR physiotherapist OR physiotherapists OR “physician assistant” OR “physician assistants” OR physician OR physicians OR surgeon OR surgeons OR anesthetist OR anesthetists OR clinician OR clinicians OR doctor OR doctors) | 2,095,605 |
| #3 Combined | #1 or #2 | 2,381,512 |
| #4 Microlearning | TS=((microlearning OR "micro learning" OR “micro-learning” OR microteaching OR "micro teaching" OR “micro-teaching” OR microtraining OR “micro training” OR “micro-training” OR “nanolearning” OR “nano-learning” OR “on-demand learning” OR “on-demand training” OR “on-demand learning” OR microlecture OR microlectures OR "micro lecture" OR "micro lectures" OR microformat OR microfeedback OR “micro feedback” OR “micro content” OR microcontent OR “micro feedback” OR microeducation OR “micro education” OR “micro-education” OR “micro-lesson” OR “micro-lessons” OR microskill OR "micro skill" OR microskills OR "micro skills" OR microformat OR microformats OR "micro format” OR "micro formats" OR “micro-courses” OR minicourse OR “mini-learning” OR “mini-lesson” OR “mini-lessons” OR "just-in-time learning" OR "just-in-time training" OR “just-in-time teaching” OR “micro-intervention” OR “micro intervention” OR “micro-interventions” OR “micro interventions” OR "mlearning" OR "m-learning" OR “bite-sized learning” OR “learning nuggets” OR “learning on the go”) OR ((teaching OR teach OR teaches OR learn OR learning OR learns OR e-learning OR elearning) AND ("cell phone" OR cellphone OR "cell phones" OR cellphones OR “mobile application” OR “mobile app” OR “mobile applications” OR “mobile apps” OR "text messaging” OR texting OR "text message" OR "text messages" OR “smart phone” OR smartphone OR “smart phones” OR smartphones OR iphone OR iphones OR android OR androids OR ipad OR ipads OR tablet OR tablets OR mobile OR mobiles OR ubiquitous OR apps OR app OR podcast OR podcasts OR podcasting OR "short message service" OR blog OR blogs OR blogging OR "instant messaging" OR "instant message" OR “instant messages” OR forum OR forums OR “discussion board” OR “discussion boards” OR “short video” OR “short videos” OR “short audio” OR infographic OR “social media” OR chunking OR snippets OR gamified OR gamification))) | 143,118 |
| #5 Combined | #3 AND #4 | 11,273 |
| #6 Study Type Filter | Web of Science User Interface: Limit Document Type to “Article” and “Review Article” | 9,552 |

*Note*: Although the Web of Science user interface filter was limited to “Article” and “Review Article,” document-type metadata in the final dataset included a small number of records labeled as proceedings papers or book chapter following export and metadata verification.

**Table S2.** Summary of bibliometric analyses and mapping procedures.

The following table summarizes the bibliometric analysis workflow and mapping procedures used to generate the network visualizations and citation-based outputs reported in the manuscript.

| **Analysis Type** | **Unit of Analysis** | **Software** | **Counting / Metric** | **Normalization** | **Visualization Rule** | **Included Items** |
| --- | --- | --- | --- | --- | --- | --- |
| Co-authorship network | Authors | VOSviewer | Full counting | Association strength | Minimum documents per author = 1; connected items only | 32  authors |
| Co-authorship network | Institutions | VOSviewer | Full counting | Association strength | Minimum documents per institution = 1; connected items only | 39 institutions |
| Co-authorship network | Countries | VOSviewer | Full counting | Association strength | Minimum documents per country = 1; connected items only | 27  countries |
| Co-occurrence network | Author keywords | VOSviewer | Full counting | Association strength | Minimum occurrence threshold = 2; connected items only | 67 keywords |
| Descriptive bibliometric analysis | Dataset-level overview | Biblioshiny (Bibliometrix) | Frequency-based summary | N/A | N/A | 560 documents |
| Citation structure analysis | Locally cited references | Biblioshiny (Bibliometrix) | Local citation counts | N/A | Ranked output | 13,229  cited references |

*Note.* Thresholds were applied in VOSviewer to reduce network complexity and improve interpretability. Minimum occurrences were set at 2 for author keywords, and minimum documents were set at 1 for authors, institutions, and countries. Final visualizations included only connected items retained after software-based selection procedures. Biblioshiny analyses were used to generate descriptive and citation-based summaries from the cleaned Web of Science dataset.


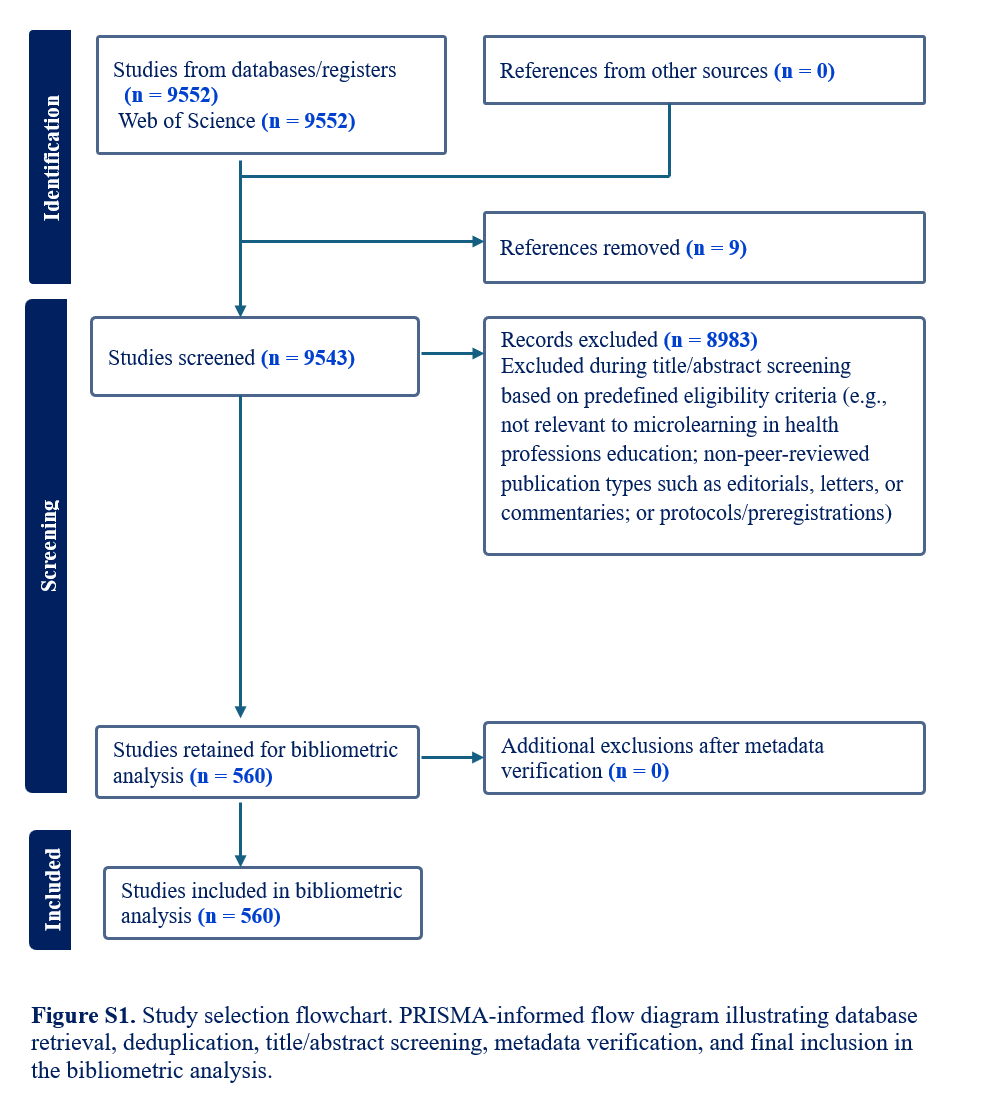


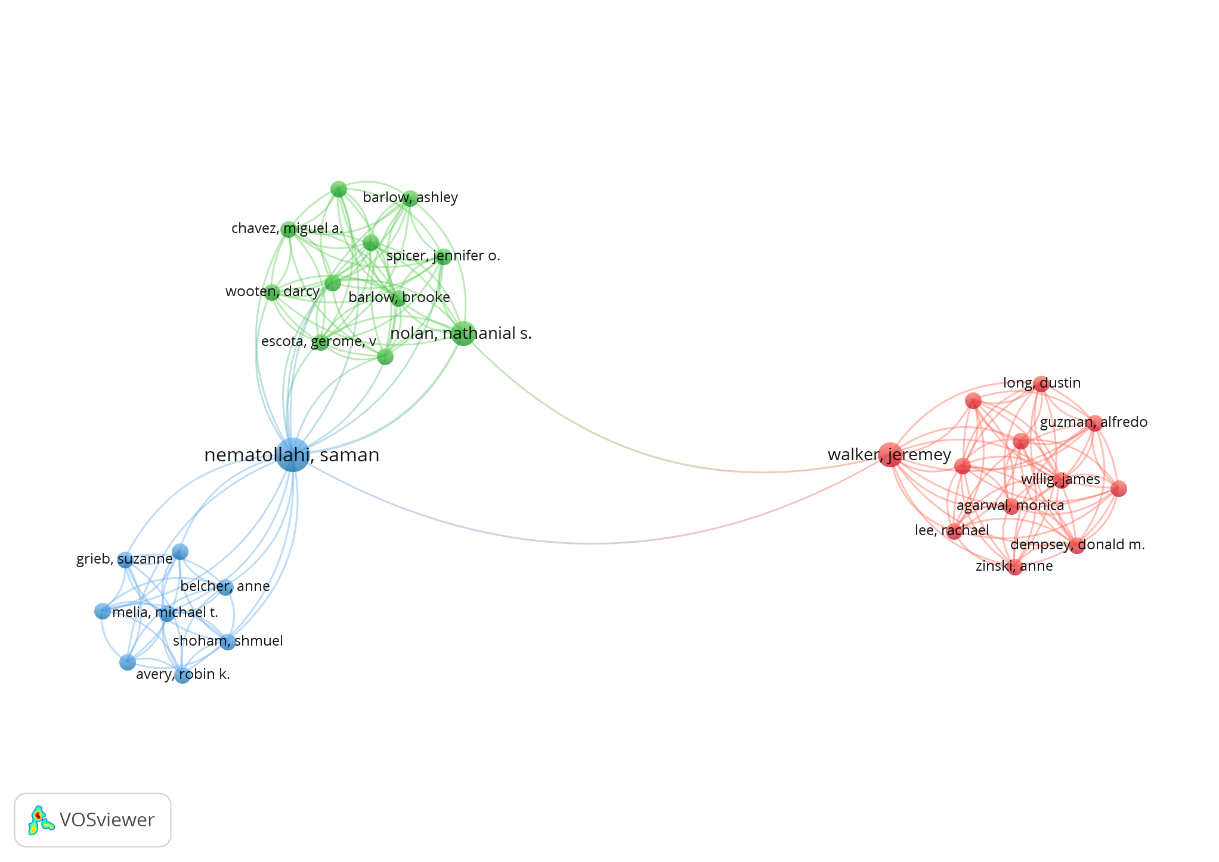


**Figure S2.** Author co-authorship network. Node size reflects publication output, and line thickness indicates collaboration strength (TLS). Proximity between nodes represents co-authorship frequency, and color clusters denote collaborative author groups. Thresholds were applied to reduce network complexity, and only connected items were included in the visualization.

*
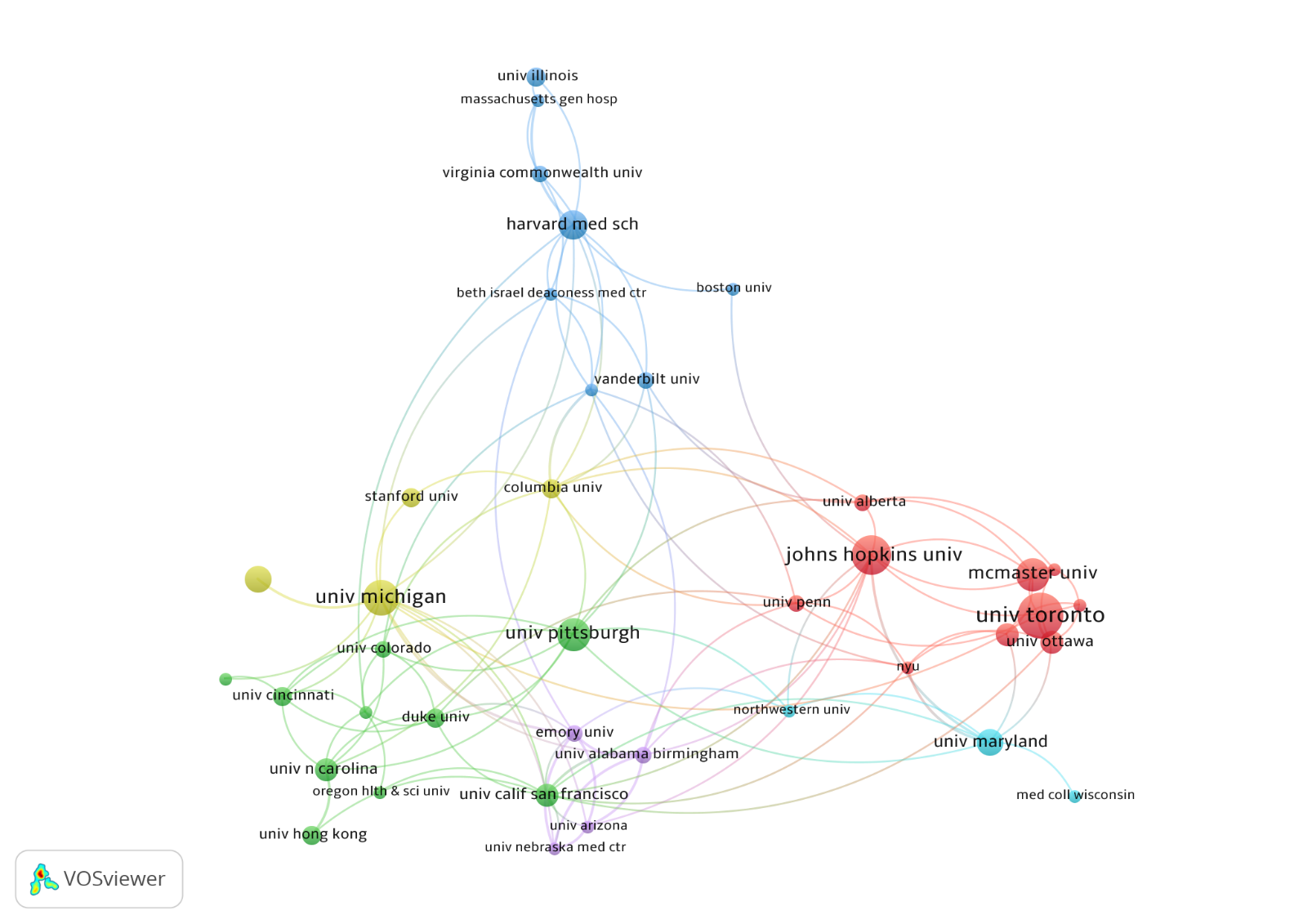
*

**Figure S3.** Institutional co-authorship network. Node size reflects total publications, and line thickness indicates collaboration intensity (TLS). Proximity between nodes represents co-authorship relationships, and color clusters denote collaborative groupings among institutions. Thresholds were applied to reduce network complexity, and only connected items were included in the visualization. Institutional labels reflect source metadata and may vary based on affiliation indexing and normalization.
